# Survey of Experts on Current Endothelial Keratoplasty Techniques

**DOI:** 10.4172/2155-9570.1000608

**Published:** 2016-10-27

**Authors:** Winston Chamberlain, Ariana Austin, Mark Terry, Bennie H Jeng, Jennifer Rose-Nussbaumer

**Affiliations:** 1 Department of Ophthalmology, Oregon Health Sciences University, Portland, USA; 2 Francis I. Proctor Foundation, University of California, San Francisco, San Francisco, USA; 3 Devers Eye Institute, Portland, USA; 4 Department of Ophthalmology and Visual Sciences, University of Maryland School of Medicine; Baltimore, USA; 5 Department of Ophthalmology, University of California, San Francisco, USA

**Keywords:** Cornea, Endothelial keratoplasty, DMEK, DSEK, UT-DSEK

## Abstract

**Objective:**

To survey cornea specialists’ opinions on different endothelial keratoplasty techniques and to gauge the perceived need for and utility of a randomized controlled trial (RCT) comparing them.

**Methods:**

A short survey was distributed to a group of cornea specialists at the Endothelial Keratoplasty Group meeting at the American Academy of Ophthalmology meeting in November 2015.

**Results:**

Thirty-three of 80 practicing surgeons present at the EKG meeting participated in the survey, yielding a response rate of 41%. Ninety-seven percent (n=32) of our respondents reported performing Descemet’s Stripping Endothelial Keratoplasty (DSEK) regularly, and 70% reported having performed Descemet’s Membrane Endothelial Keratoplasty (DMEK) at least once (n=23). While most respondents (n=26, 79%) thought there was at least some evidence that DMEK is superior to DSEK in terms of visual acuity, there was less certainty about comparing ultrathin-DSEK (UT-DSEK) to DMEK with 48% (n=16) thinking there was at least some evidence of DMEK’s superiority, 6% (n=2) thinking there was at least some evidence of UT-DSEK’s superiority, and 30% (n=10) unsure. Seventy-two percent (n=23) of respondents thought an RCT comparing visual acuity outcomes in UT-DSEK versus DMEK would be at least moderately beneficial, and 82% (n=27) reported they were at least moderately likely to change their EK technique based on the results of said RCT.

**Conclusion:**

There is substantial interest in an RCT comparing visual acuity outcomes in UT-DSEK versus DMEK.

## Introduction

Corneal transplantation has evolved rapidly in recent years. Lamellar keratoplasty to replace diseased endothelium has led to faster recovery times, fewer complications, and better visual acuity outcomes [1]. Currently, Descemet Stripping Endothelial Keratoplasty (DSEK) is the most commonly performed endothelial keratoplasty (EK) procedure because of its relative ease and good outcomes [2]. Newer EK techniques such as Descemet’s Membrane Endothelial Keratoplasty (DMEK), where Descemet’s membrane alone is transplanted, have the potential to further improve visual acuity outcomes and decrease rejection rates [3–7]. However, donor preparation, increased intraoperative times, and problems with donor attachment in DMEK are all important limitations [8–10].

Ultrathin DSEK (UT-DSEK) employs the same surgical techniques as traditional DSEK techniques but with thinner donor grafts. This procedure may have similar results to DMEK but without the technical difficulties [11]. However, UT-DSEK has not been well defined in the literature with no consensus regarding what graft thickness constitutes ultra-thin. Moreover, some papers describe the tissue thickness measurement pre-operatively and some post-operatively [12,13]. Several prospective series show similar visual outcome results and rates of immunologic rejection between UT-DSEK and DMEK, however comparisons between studies are difficult [11,14–17].

There is little information about current opinions of cornea specialists on the benefits of UT-DSEK versus DMEK. It is also unclear how cornea specialists define UT-DSEK and how often they are performing it relative to other EK techniques. In this study we assess cornea specialists’ practice patterns and opinions on the different EK techniques.

## Methods

A paper survey was distributed to conference attendees at the Endothelial Keratoplasty Group (EKG) meeting at the American Academy of Ophthalmology conference in November 2015. All participants were ophthalmologists. Participation was voluntary and no personal information or identifiers were collected. The survey was designed and created using Research Electronic Data Capture (REDCap). The survey was collected on the same day that it was distributed.

The survey was made up of 22 questions about endothelial keratoplasty (Appendix A). The survey collected information on demographics, practice characteristics, and physician characteristics. For both DSEK and DMEK, respondents were asked if they perform each technique, and if they do, why they chose to adopt it. Respondents were then asked to weigh the current level of evidence to support the superiority in visual acuity outcomes of DSEK, DMEK, and UT-DSEK. Finally respondents were asked about their thoughts on a randomized controlled trial (RCT) comparing DMEK and UT-DSEK, and if the results from an RCT would impact their choice of surgical technique.

Responses were analyzed using Stata 14.0 (StataCorp, College Station, TX). Institutional Review Board approval was obtained from the University of California, San Francisco Committee on Human Research. This study adhered to all federal and state laws, as well as the Declaration of Helsinki.

## Results

We received a total of 33 responses to the survey, for a response rate of 51% of practicing surgeons present. Table 1 outlines the study participant demographics and EK practice patterns. Respondents were mostly from the United States (n=29), with additional respondents from Argentina (n=1), Brazil (n=1), Colombia (n=1), and Malaysia (n=1). All were Cornea and Refractive specialists, and all reported performing EK routinely. Respondents were mostly in private practice (n=20, 61%), with the remainder practicing at a university hospital (n=12, 36%), government hospital (n=1, 3%), or other hospital (n=1, 3%) (one participant reported working both in private practice and at a government hospital). Respondents had a median of 25 years in practice (IQR 10, 29) and median of 8 years performing EK (IQR 5, 10). The median number of EK surgeries performed each month was reported to be 6 (IQR 3, 10).

Respondents were asked to estimate what percentage of their corneal transplants for isolated endothelial disease were DSEK, UT-DSEK, DMEK, and penetrating keratoplasty (PKP). Median values were 33% (IQR 10%, 53%) for DSEK, 0% (IQR 0%, 0%) for UT-DSEK, 33% (IQR 0%, 75%) for DMEK, and 5% (IQR 0%, 20%) for PKP.

Ninety-seven percent of respondents (n=32) reported performing DSEK. The median thickness of DSEK grafts was reported to be 117.5 μm (IQR 100 μm, 125 μm). Respondents were asked about why they adopted DSEK, and 97% (n=32) reported that they adopted DSEK because of its superiority to PKP. Other reasons listed for adopting DSEK included wanting to remain on the cutting edge of surgery (n=9, 27%) and because of its superiority to other EK techniques (n=6, 18%). Respondents were asked about what graft thickness they considered to be the threshold for UT-DSEK. The most common response was <100 μm (n=18, 56%), followed by <80 μm (n=7, 22%). One person each reported their UT-DSEK threshold was <150 μm, <125 μm, and <120 μm (3% each), and 13% (n=4) did not know how to define it.

Seventy percent of respondents (n=23) reported performing DMEK in their practice. The most common reason for adopting DMEK was its superiority to other EK techniques (n=21, 91%). Other reasons listed included its superiority to PKP (n=15, 65%), and wanting to remain on the cutting edge of corneal surgery (n=12, 52%). Most respondents learned DMEK by taking a DMEK course (n=15, 65%), while some were self-taught (n=6, 26%) and the minority learned during fellowship training (n=4, 17%).

**Figure 1:**
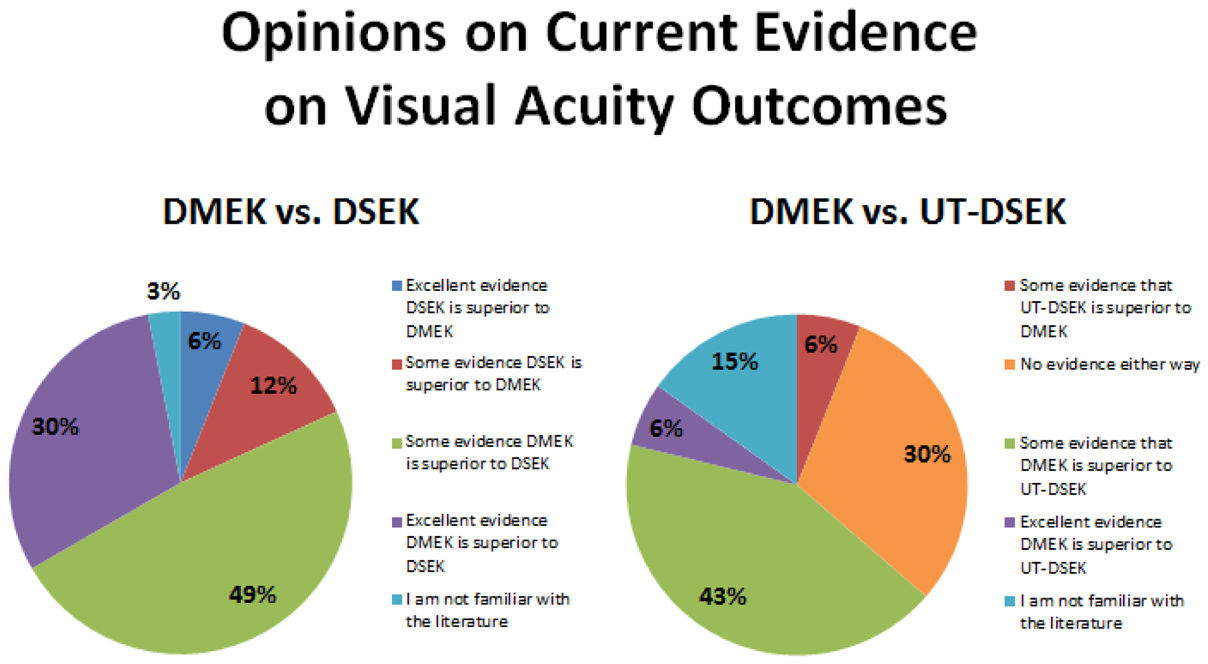
Pie charts showing opinions on current evidence of visual acuity outcomes in DMEK versus DSEK and DMEK versus UT-DSEK.

Respondents were asked to weigh the current level of evidence in the literature regarding visual outcomes for DMEK versus DSEK (Figure 1). The vast majority (n=26, 79%) thought there was at least some evidence that DMEK is superior to DSEK, with 30% (n=10) reporting that there is excellent evidence DMEK is superior to DSEK. When asked about the current level of evidence in the literature concerning visual outcomes for DMEK versus UT-DSEK, there was less of a consensus. Forty-eight percent (n=16) of respondents thought that there was at least some evidence DMEK is superior to UT-DSEK, while 30% (n=10) thought there was no evidence either way or 6% (n=2) thought there was some evidence that UT-DSEK is superior to DMEK.

**Figure 2:**
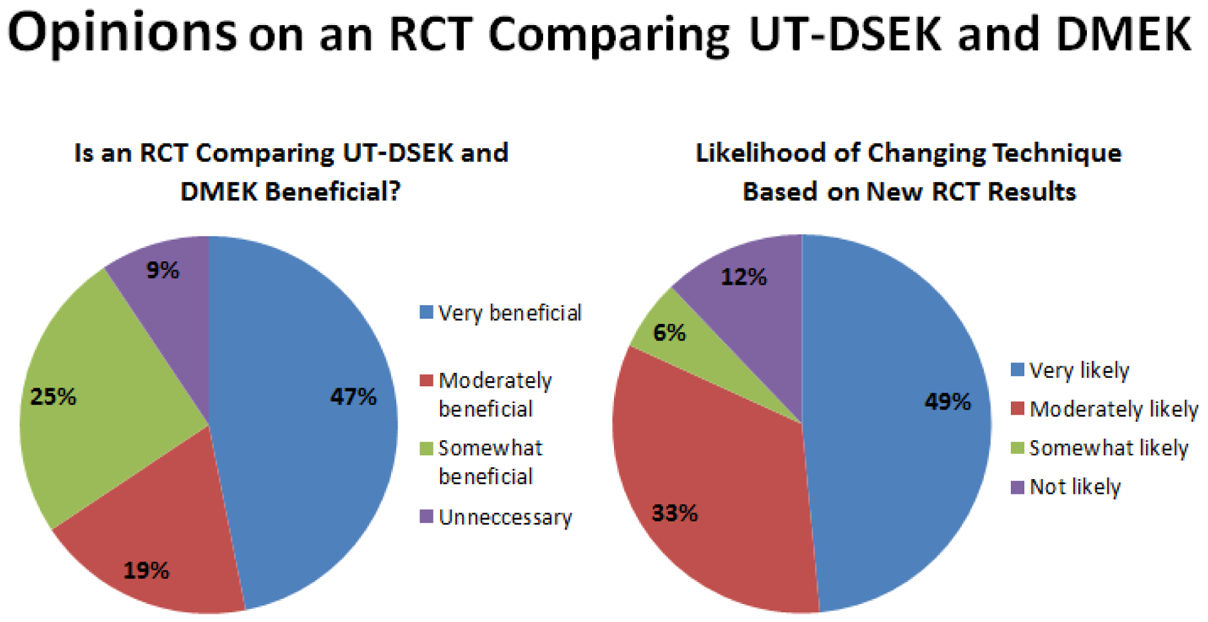
Pie charts showing opinions on perceived need for and utility of an RCT comparing UT-DSEK and DMEK.

When asked if they thought that an RCT comparing UT-DSEK and DMEK would be beneficial, 72% (n=23) reported that it would be at least moderately beneficial, with 47% (n=15) reporting that it would be very beneficial (Figure 2). The remainder reported that they thought an RCT would be somewhat beneficial (n=6, 19%), with only 9% (n=3) reporting an RCT was unnecessary. Eighty-two percent (n=27) of respondents reported that they were at least moderately likely to change their EK technique based on the results of an RCT, with 48% (n=16) reporting they were very likely to change techniques. Four people (12%) reported being unlikely to change their EK technique based on new RCT results.

## Discussion

In this study we report experienced corneal surgeons’ practice patterns and opinions regarding EK techniques. While most respondents thought that there was at least some evidence that DMEK is superior to DSEK with regard to visual acuity, the majority of EK surgeons in our study were performing approximately equal numbers of DSEK and DMEK. This may be due to the fact that DMEK is difficult to perform in eyes with prior glaucoma or retinal surgery, or severe corneal edema. Additionally, many of these surgeons may still be on the DMEK learning curve, reserving DMEK for the more routine cases of Fuchs endothelial dystrophy or bullous keratopathy. As expected, PKP was performed much less frequently.

There was less certainty about the superiority of DMEK compared with UT-DSEK. Nearly one third of respondents thought that there was no evidence in the literature to support one technique over the other. Only six of our respondents (18%) reported performing any UT-DSEK. This is likely due to conflicting opinions on the definition of UT-DSEK in the literature [12,13,17]. Based on our survey results, most EK surgeons would agree that UT-DSEK grafts must be less than 100 μm, however, we did not ask whether this measurement was preoperative or post-operative. The vast majority thought an RCT comparing visual acuity outcomes in UT-DSEK and DMEK would not only be valuable, but would change their clinical practice. This finding is especially noteworthy considering the high percentage of surgeons performing DMEK in this group, and thus the likely preference of our sample for surgeons who prefer DMEK.

Strengths of this study include our excellent response rate and the fact that the vast majority of our respondents were experienced corneal surgeons who are regularly performing all types of EK. Limitations of this study include the smaller sample size, and the highly specialized group of respondents, which may not be reflective of the broader community of corneal specialists or other ophthalmologists.

In this survey we demonstrate community equipoise among experienced corneal surgeons regarding outcomes of UT-DSEK versus DMEK. There is substantial interest in an RCT comparing visual acuity outcomes between these techniques, even among this group of surgeons, most of whom perform DMEK.

## Figures and Tables

**Table 1:** Physician and Practice Characteristics of Respondents.

Location	N	
United States	29	
Argentina	1	
Brazil	1	
Colombia	1	
Malaysia	1	
**Physician Characteristics**	**Median**	**IQR**
Years in practice	25	10, 29
Years performing EK	8	5, 10
EK surgeries performed per month	6	3, 10
**Percentage of EK’s Performed That Are:**	**Median**	**IQR**
DSEK	33	10, 53
UT-DSEK	0	0, 0
DMEK	33	0, 75
PKP	5	0, 20

IQR: Interquartile Range; EK: Endothelial Keratoplasty; DSEK: Descemets Stripping Endothelial Keratoplasty; UT-DSEK: Ultrathin Descemets Stripping Endothelial Keratoplasty; DMEK: Descemets Membrane Endothelial Keratoplasty; PKP: Penetrating Keratoplasty
